# Adult acne versus adolescent acne: a narrative review with a focus on epidemiology to treatment^☆^

**DOI:** 10.1016/j.abd.2022.01.006

**Published:** 2022-10-14

**Authors:** Ömer Kutlu, Ayşe Serap Karadağ, Uwe Wollina

**Affiliations:** a Department of Dermatology and Venereology, School of Medicine, Tokat Gaziosmanpasa University, Tokat, Turkey; b Department of Dermatology and Venereology, Memorial Ataşehir Hospital, İstanbul, Turkey; c Department of Dermatology and Allergology, Academic Teaching Hospital Dresden, Dresden, Germany

**Keywords:** Acne, adult, Adolescent acne

## Abstract

Acne vulgaris is one of the most common chronic inflammatory diseases and is characterized by papules, pustules, comedones, and nodules. Although adolescence is the preferential age group, acne may affect various age groups. Acne shares different properties in adults and adolescents. These differences extend from epidemiology to treatments. Increased awareness of these two subtypes will allow for better management of the disease. In this review, the authors examined all aspects of acne in adults and adolescents under the light of current literature.

## Introduction

Acne vulgaris is one of the most common chronic inflammatory diseases in the world and is characterized by papules, pustules, comedones, and nodules. Although adolescence is the preferential group, acne may affect various age groups.^1^, ^2^ The term “adolescent acne” is determined to age between 10‒19 years. However, this term is somewhat misused for patients between 19‒24 years of age. According to WHO, the terms “young people” cover the age range 10‒24 years, hence, the authors suggest patients who developed acne between 10‒24 years should be referred to as “young acne” while patients who developed acne between 10‒19 years should be referred to as “adolescent acne”. Previous studies have reported that up to 9.3% of acne occurs after 25 years of age.^3^ Acne > 25 years is called post-adolescent or adult acne.^2^ Similarly, the authors believe that the term “adult acne” can be more appropriate than “post-adolescence” since post-adolescence suggests individuals above 19 years of age.

Adult acne is classified into three groups within itself. If it starts in adolescence period and continues, it is called “persistent acne” while if it develops for the first time after the age of 25 it is called “late-onset acne”. Lastly, “relapsing acne” is used for acne which heals and continues in attacks from the young to adult age.^4^, ^5^ However, in many studies, acne has been noted in two classes including persistent acne and late-onset acne. Persistent acne has been reported as the most common type of adult acne responsible for 73.2%‒82% of cases.^2^, ^6^

Adult acne includes various pathophysiological factors, different clinical features, and treatment approaches compared with adolescent acne ​Table 1​ ​​. In this section, common features and differences between both types of acne will be discussed.

**Table 1:** The characteristics of adult acne and adolescent acne.

	Adolescent Acne	Adult Acne
**Age**	10‒19 years^a^	> 25 years
**Gender**	More common in males	More common in females
**Severity**	Mild: comedonal type	Usually mild inflammatory-papular type
	Severe: nodulocystic type	
**Localization**	Face involvement: cheek	Face involvement: cheek, chin, mandibular
	Truncal involvement: common	Truncal involvement: rare
**Lesion type**	Comedones	Inflammatory papulopustular lesions
**Comedones**	Common	Rare, but more common in smokers
**Inflammatory papules**	Common	Very common
**Cyst**	Can be exist	Rare
**Scar**	Depend on severity	Common
**Sebum production**	Increased	Increased
**Microbial flora**	*C. acnes*	*C. acnes*
**Hormonal involvement**	Can be exist	Very common
**Response to treatment**	Good	Often resistant/relapse frequent

a Acne occurs at age of 10‒24 years should be referred to as “young acne”.

## Epidemiology

Acne vulgaris tends to be more severe in males while its frequency increases in females after the age of twenty. Although previous reviews have reported that acne is more common in females than males, a systematic epidemiologic review suggested that this is a controversial issue.^7^, ^8^, ^9^

In a cross-sectional epidemiologic study, the presence of adolescent acne has been reported among 27.9% of boys and 20.8% of girls.^10^ On the other hand, adult acne is preferentially observed in women. In a study with 280 patients, 82.1% of the patients with adult acne were women.^6^ In another study, late-onset acne was found in 97.3% of women.^2^

## Etiopathogenesis

Abnormal follicular keratinization, although it is not always present in increased sebum production, qualitative differences in sebum composition such as squalene, triglycerides, and wax/cholesterol esters, presence of *Cutibacterium acnes* (*C. acnes*), and inflammation play a fundamental role in the pathogenesis of acne vulgaris in both adolescent and adult acne. Of note, there is no hypercolonization of *C. Acnes* in acne patients, but there are particular differences in *C. acnes* phylotypes between healthy and acne-prone skin, such as type IA and IC strains identified by multilocus sequence typing.^11^, ^12^

The distinction between adolescent and adult acne pathogenesis remains unclear while studies on the causes of adult acne are still in progress. Hormones, colonization by resistant bacteria, cosmetics, drugs, and chronic stress are possible etiological factors. However, certain additional factors are particularly considered as the possible mechanism of adult acne.

Although there are no differences in the bacterial colonization between adolescent and adult acne, chronic stimulation of the resistant strains of *C. acnes* in the innate immune system may cause inflammatory lesions of acne in adults.^13^, ^14^, ^15^, ^16^ A predominance of adult acne in women may be related to hormonal factors. Although some studies reported that endocrinologic conditions such as hirsutism, premenstrual exacerbation, and androgenetic alopecia are more common in adult female acne, hyperandrogenism markers are found to be high in very few patients in laboratory tests.^2^, ^6^ Nevertheless, it has been reported that even in the upper limits of normal values, DHEA-S may stimulate IL-2 production and hereby the Th1 system.

Despite the fact that the frequency of polycystic ovaries has been reported as high as 52%‒82% in adult female acne, the hormonal profile is often not compatible with Polycystic Ovarian Syndrome (PCOS).^17^ The exact effect of hormonal factors on adult acne is controversial. In addition to studies reporting high serum levels of testosterone and Dehydro-Testosterone (DHT),^18^ there are also studies reporting that hormonal levels are normal.^19^, ^20^ These results suggest that end-organ hypersensitivity may be more important than high serum androgen levels in the occurrence of acne.^21^ An increased sensitivity or intracrine metabolism of androgens in the sebaceous glands and potent androgen metabolites in the skin are among the possible mechanisms.^19^, ^21^ It is also considered that a certain number of follicles are susceptible to acne, and these follicles show different levels of sensitivity to circulating androgen hormones.^6^

Sebocytes and keratinocytes have an enzymatic system that is able to produce testosterone and DHT. Hyperactivity and abnormal activity are observed in enzymes that result in the metabolization of androgenic hormones such as 5-alpha reductase, 3-beta-OH steroid dehydrogenase, and 17-OH steroid dehydrogenase. These enzymes increase the peripheral turnover of pro-hormones (DHEAS, androstenedione, and testosterone) that lead to the formation of more potent androgenic hormones (testosterone and DHT).^22^, ^23^ In this context, DHT is 5‒10 times more potent than testosterone.^24^ Production of sebum can also be triggered by neuropeptides, stress, and other hormones such as melanocortins and Corticotropin-Releasing Hormone (CRH).^25^ Histamine, vitamin D, retinoids, and Insulin-like Growth Factor 1 (IGF-1) have been determined as adjusting factors of sebum production.^5^

## Triggering factors

Acne can be affected by numerous external and internal factors. Diet, premenstrual exacerbation, hyperhidrosis, stress, smoking, genetic predisposition, drugs, cosmetics and moisturizers, seasonal factors, and hormonal disorders are among the triggers that play an important role in the pathogenesis of acne.^2^, ^6^, ^26^, ^27^, ^28^ Recently protective facial masks have been associated with facial acne (mascne).

### Body mass index and dietary factors

There are numerous studies that reported insulin resistance and a Western diet associated with the existence and flare-up of acne.^26^, ^29^ Although most studies revealed that Body Mass Index (BMI) may have a role in the etiopathogenesis of acne, there are contradictory results regarding this issue.^26^, ^30^, ^31^ In a large population-based study, it was found that being overweight and obesity are inversely related to acne in a dose-dependent manner.^32^ Furthermore, another study reported a negative association between BMI and acne lesion counts found in women with adult acne.^33^ On the other hand, other studies reported that obesity and overweight may induce the risk of acne vulgaris in adolescents and young adults,^30^, ^34^ but others failed to demonstrate any association between acne and BMI.^26^, ^35^

Although there are conflicting results on the relationship between BMI and acne in both adolescence and adulthood, it has been suggested that obesity in PCOS results in insulin resistance leading to a severe form of hyperandrogenism and acne by elevating free and bioavailable testosterone levels.^36^

Previous studies revealed that insulinotropic milk/dairy products and glycemic load consisting of hyperglycemic carbohydrates increase the signaling of insulin/IGF-1 while decreasing the levels of Insulin-like Growth Factor Binding Protein 3 (IGFBP-3).^37^, ^38^ IGFBP-3 inhibits apoptosis and stimulates cell proliferation by binding to retinoid X receptor-α.^39^, ^40^ On the other hand, IGF-1 decreases nuclear levels of the metabolic forkhead box class O transcription factor 1 (FoxO1), which cause activation of the mammalian Target of Rapamycin Complex 1 (mTORC1). Increasing expression of the mTORC1 leads to sebaceous gland hyperproliferation, lipid synthesis, and hyperplasia of keratinocytes that result in acne.^41^ In this context, recent epidemiological evidence and controlled studies on the relationship between acne and diet have shown an association between the occurrence and severity of acne and certain food consumption.^26^ Penso et al. reported consumption of milk, sugary beverages, and fatty and sugary products may be related to current acne in adults.^42^ Karadağ et al. reported white sugar, dairy products, ice cream, and white bread could increase acne severity in patients under 25 years of age. An inverse proportion has been reported between whole wheat bread, fish, and legumes and acne development.^31^ In addition, Kutlu et al. reported in the 12‒18 age group grapes consumption were significantly higher in the healthy group than in the acne group and acne severity was significantly lower in the group that more consuming tangerine per week.^26^ Chocolate, french fries, cola, pizza, and dry foods are among the most implicated agents of acne regardless of age.^42^, ^43^, ^44^, ^45^

### Premenstrual flare-up

Premenstrual flare is associated with both adolescence and post-adolescence acne. According to previous studies, a premenstrual flare of acne seems to be higher in adult female acne (78%) than in adolescent female acne (52.8%).^31^, ^46^ The occurrence of acne in the premenstrual period is explained by the increased water content of the pilosebaceous unit causing sebaceous duct orifice blockage. This exacerbation may also be relevant to the drugs such as progesterone-derived oral contraceptives.^47^, ^48^, ^49^

### Hyperhidrosis

It has been considered that hyperhidrosis may induce acne. A previous study reported that 15% of patients experience an aggravation of acne after sweating, with ductal hydration believed as a responsible factor. In a study conducted with 537 students in Bangkok, more than half of the patients stated that acne is worsened by sweating and exposure to hot weather.^50^ However, in a randomized single-blind pilot study, Short et al. reported no relationship between exercise-induced sweat on truncal acne in boys.^51^ Dermcidin is one of the antimicrobial peptides released from eccrine sweat glands and sebaceous glands. The previous study revealed that a low level of dermcidin concentration in sweat glands may permit the proliferation of P. acnes in the pilosebaceous unit.^52^ Excessive sweating may cause dysregulation of the dermcidin that can trigger acne. The exact effect of hyperhidrosis on both acne types should be elucidated by further studies.

### Stress

Emotional stress is an important triggering factor for both types of acne. Stress causes excoriations and skin-picking in patients with acne that increase the risk of inflammation, scarring, and hyperpigmentation of lesions.^53^ The fact that stress causes stimulation of androgen hormones makes it more important in adult acne. Emotional stress may also induce cortisol levels by altering the adrenal-pituitary axis. The previous studies reported emotional stress is a triggering factor for 25.7%‒71% of patients with adult acne.^2^, ^6^ Hormonal exacerbations associated with menstruation may attribute the increased stress in females.

### Smoking

There are few available studies on a potential correlation between acne and smoking. Most studies support that cigarette smoking may prone to acne.^54^, ^55^, ^56^ Pelle et al. demonstrated that cigarette smoking induces peroxidation in human skin which may lead to changes in sebum composition.^57^ Capitanio et al. reported a strong correlation between smoking habits and the high prevalence of non-inflammatory adult female acne. In this context, they postulated that smoking may be a more important factor than emotional stress in patients with female adult acne.^58^ On the other hand, smoking was interestingly found significantly associated with lower acne prevalence in adolescent girls.^59^ In conclusion, given the high prevalence of smoking among adults, the authors believe that the effect of cigarette smoking on acne is more important in adults than in adolescent acne. Further studies are required to elucidate the exact effect of cigarettes on the occurrence and severity of acne in both acne types.

### Genetic predisposition

The presence of acne history in first-degree relatives is common in both adult and adolescent acne what supports a genetic predisposition.^29^, ^60^ Khunger et al. reported that 38.8% of the patient with adult acne have at least one first-degree family history of acne while other studies reported a higher incidence of 50%‒70.9%. In addition, a family history of acne has been shown as an indicator of the higher risk of relapse in adult female acne.^2^, ^6^, ^60^, ^61^ On the other hand, an epidemiological study from a French school performed among 913 adolescents reported that the history of acne in the mother and father was 25% and 16% in the acne group respectively while it was 14% and 8% in the group without acne.^10^

Karadağ et al. reported a significantly higher percentage of early-onset acne in young acne among with a family history of acne, a similar result was found by Suh et al. in acne patients regardless of their age.^31^, ^62^

In another study, the prevalence of moderate to severe acne in high school pupils who have positive family history has a 2.3 times higher risk than those without a family history of acne.^63^

The confocal microscopy of adult acne in women revealed a larger number of follicles with enlarged infundibulum compared to control and a greater number of comedones that show that genetically susceptibly to hyperseborrhea.^64^

### Drugs

There are certain drugs that can trigger the presence of acne vulgaris. The drug-induced acne usually consists of monomorphic papular lesions that resemble rose pearls.^65^ Adults are more confronted with drug-related acne due to receiving more drugs than adolescents. The common culprit drugs in routine clinical practice are mostly corticosteroids, oral contraceptives, and vitamin B12. Isoniazid, testosterone, lithium, and certain anticancer drug are also related to drug-induced acne.^66^, ^67^ Steroids cause acne by inducing TLR-2 in the upper part of the pilosebaceous unit.^66^ Contraception with drugs containing an androgenic progestin and dermal contraceptive implants may cause acne along with other systemic symptoms (norgestrel, levonorgestrel, etonogestrel, DMPA); for this reason, more selective 3rd and 4th generation combined oral contraceptives with minimal androgenic properties may be preferred over earlier formulations.^27^, ^28^

### Cosmetics and moisturizers

The common use of cosmetics and moisturizers is a rising issue for both adolescent and adult acne in females. Adolescent girls mostly wear makeup for their acne, while adult women do for their hyperpigmentation. Cosmetic use does not take into account an etiological factor in adult acne in some reports. However, in a previous study, 22% of adult females reported that their acne was triggered by cosmetics.^6^ Cosmetic ingredients such as lanolin, isopropyl myristate, cetyl alcohol, and stearic acid possess comedogenic properties. Certain oily sunscreens commonly worn by women are also culprit agents for triggering comedonal acne.^47^

### Seasonal factors

The role of seasonal factors in triggering acne is one of the concepts discussed in the literature. It has been thought that acne improves in summer and exacerbates in winter. On the contrary, cases triggered after sun exposure and in the summer months are also reported. Along with its inflammatory effects, ultraviolet radiation may cause an increase in squalene peroxidase which has comedogenic properties. The summer season has been reported as a triggering factor for acne in 32% of adults versus 80.6% of adolescents.^6^, ^68^ However, it remains unclear whether the summer season has a role in the existence or flare-up of acne.

### Hormonal disorders

Hormonal disorders mostly trigger adult acne. The sudden onset of adult acne should be investigated in terms of virilization symptoms as well as underlying endocrine disorders. Irregular menstrual cycles (hypermenorrhea, amenorrhea, oligomenorrhea), clitoral hypertrophy, hirsutism, female pattern hair loss, late menarche (>15), or resistant and sudden-onset acne are systemic signs and symptoms of hyperandrogenism. Other features such as obesity, infertility, metabolic syndrome, and hypothyroidism need to be examined. Adrenal hyperplasia, primarily virilizing tumors, and PCOS should be investigated in patients who have abnormal laboratory results.^29^ It has been recommended to investigate hormonal disorders in adult acne. However, the rate of hormonal disorders that can be detected in peripheral blood is variable, and in one study, a high testosterone level suggesting hyperandrogenism has been reported in only 3.04%.^6^ Hormonal examinations are performed 3‒5 days after the onset of menstruation and free and total testosterone, DHEA-S, androstenedione, 17-alpha hydroxy-progesterone, SHBG (sex hormone binding protein), prolactin, and cortisol levels should also be checked. Mild to moderate increase of anti-mullerian hormone, DHEAS, and low levels of SHBG can be seen in the laboratory tests as signs of peripheral hyperandrogenism in adult female acne.

## Clinical characteristics

Adult acne has been mostly observed as inflammatory papulopustular lesions in clinical practice and occurs gradually and in general remains in a mild-moderate course, unlike adolescent acne. Previous studies have reported that acne ranging from mild to moderate is seen in about 61%‒85% of adult female acne.^6^, ^60^ In a prospective multicenter, cross-sectional study it has been reported that acne during pregnancy resembles the features of female adult acne. The severity of facial acne, truncal acne, and hirsutism has been reported higher in the third trimester.^69^ Comedones are rarely seen in adult acne. However, large cyst-like comedones can occasionally be scattered all over the face in adult female acne (Pyoderma faciale). It has been suggested that smoking is an important trigger in such cases.^70^ On the other hand, adolescent acne usually starts with comedones, and acneiform lesions are seen in a wide spectrum, from mild inflammatory lesions to severe nodulocystic lesions.^71^, ^72^

Adult female acne is characterized by deep-seated, small nodules on the chins and cysts in the *U* zone (periorbital region, jaw, and anterior cervical region). However, both inflammatory and non-inflammatory lesions can also be scattered over the face.^60^ Hyperseborrhea is present in approximately 70% of the patients while flaring up before menstruation was reported in 80%. In addition, facial involvement is common while truncal acne is less observed.^60^, ^73^ In a previous study on adult acne, it has been reported that cheek involvement (81%) was the most common site on the face followed by the chin (67%), mandibular area (58.3%), forehead (51.7%), and nose (18.3%). Truncal involvement is reported in only 2.1% of the patients.^6^ On the other hand, adolescent acne usually presents both inflammatory papulonodular lesions, cysts, and comedonal lesions on the face. The most common site on the face is the T-zone which includes the forehead, nose, and cheeks.

Scar formation is a more common feature of adult acne than adolescent acne. The fact that adult acne includes more inflammatory lesions and is resistant to treatment increases the risk of scar formation. In this regard, it has been reported acne scarring develops in 20%‒76.4% of adult acne.^3^, ^6^, ^74^ Dreno et al. reported that acne scaring in the family history is 28.8% in adult female acne.^60^ Furthermore, Khunger et al. reported macular scars, ice pick, rolling, atrophic, and keloid scars are the most common type of scaring in patients with adult female acne.^6^

## Differential diagnoses

Adolescent acne does not usually require differential diagnosis while adult acne can somewhat be difficult to diagnose. The differential diagnoses can be sorted as papulopustular rosacea, folliculitis, syringoma, milia, Demodex folliculitis, and Pityrosporum folliculitis. It may be difficult to distinguish perioral dermatitis which is located on the chin from adult female acne due to its localization. Skin-picking associated with obsessive-compulsive disorder occasionally may cause difficulties in diagnosis, with inflammatory lesions and scarring.

## Treatment

### Pre-treatment evaluation

In the treatment of acne, a holistic approach along with pharmacological treatment is crucial. Age, gender, pregnancy status, previous treatments, and the patient’s expectation of treatment should influence treatment decisions. The duration of the lesions, cosmetics, systemic drugs, and hormonal and herbal treatments should be interrogated.

Adult women who have acne lesions, especially on the chin and cheek have to be examined for hyperandrogenism signs and symptoms and the hormonal profile should be investigated if they present irregular menses and hirsutism with or without signals of hyperandrogenism. Triggering factors (sun exposure, cold, sweating, smoking, nutrition, sleeping habits, diet, sports activities) should be questioned and the patient should be kept away from suspicious factors. Medical treatment has to consider skin type, the extent of the lesions, truncal location, severity, scarring, and post-inflammatory hyperpigmentation. In addition, anti-acne medications should be explained to the patient. Among topical treatments especially retinoids and benzoyl peroxide can cause irritant dermatitis that may develop in the first 2‒3 weeks, therefore the application may be started as an intermittent and short-contact treatment and be gradually increased the treatment duration. Since topical treatments and systemic retinoid treatment can cause dryness, peeling, and burning sensation in the skin, it is crucial to moisturize the skin with appropriate cosmetic products and to protect it from sun exposure.

### Topical treatments

The skin is more sensitive in adult acne than in adolescent acne, therefore, topical treatments are more difficult to tolerate.^74^ In general, topical treatments are adequate for mild acne. Since adult acne is more resistant to treatment and has a longer duration, it is recommended to prefer combined topical treatments or to combine systemic treatments with topical treatments in this group.^29^, ^75^ Topical retinoids, with their anticomedogenic and keratolytic effects, are the cornerstones of the treatment. Retinoids reduce pigmentation and scar formation, rebuild the papillary dermis, and show a pro-collagenase effect, therefore, it is a preferable treatment choice in adult acne where scar and pigmentation are common. Microcrystalline solutions of adapalene 0.1% and tretinoin are the most tolerated retinoids.^76^, ^77^, ^78^ It is crucial to know, that even topical retinoids are contraindicated without contraception in women during their child-bearing live-time.

Azelaic acid is one of the first-line topicals in mild to moderate adult acne. It can be used on pregnant women. It is well tolerated and reduces pigmentation by anti-tyrosinase activity. This is beneficial in adult acne since hyperpigmentation is common. Benzoyl peroxide can be used in pregnant women and can be combined with topical antibiotics or topical retinoids. Patient compliance with combined preparations is higher.^79^

### Systemic treatments

Systemic treatments are commonly administered in moderate and severe types of acne. The adult lesions on the chin can be more resistant to treatment and bear a higher risk of scarring. Systemic treatment options such as antibiotics, zinc, or hormonal treatment are preferred. Zinc is useful in reducing inflammation and can be used in pregnant women.^29^, ^80^

Hormonal treatments are possible in females if there is evidence of hyperandrogenism, premenstrual exacerbation, and hormonal symptoms. In addition, hormonal treatment can be useful even in female patients whose hormonal profile is normal but resistant to other treatments. Adjuvant antiandrogens, third, and fourth-generation oral contraceptives reduce sebum production.^81^, ^82^ Spironolactone 50‒150 mg/day is a good alternative to standard treatment for resistant adult acne, and promising results have been reported in recent years.^14^ Isotretinoin at a dosage between 0.3 to 0.5 mg/kg (European guidelines) can also be used with a minimum of 6 months duration.^83^, ^84^ Pregnancy and lactation are absolute contraindications. It is important to know that females have to perform contraception during isotretinoin treatment and up to 1 month after the treatment. A recent study proposed that the use of isotretinoin should not be a priority in adolescent patients with atherosclerotic disease and those with inflammatory bowel disease.^85^ On the other hand, there is an observational study that reported there is a possible protector effect of oral isotretinoin in inflammatory bowel disease.^86^

The current acne guidelines recommended systemic antibiotics as first-line treatment in moderate-type adolescents and young adults while isotretinoin can be used as second-line treatment. On the other hand, it has been reported that isotretinoin can be used as a first-line treatment for severe acne in both adolescents and young adults.^84^

### Cosmetics

Wearing makeup products is fairly common in adulthood. Therefore, the patients should also be recommended appropriately on skincare and cosmetological products. Patients often query whether or not to use cosmetic products. The use of appropriate products can increase the quality of life by reducing the visibility of the patient's lesions and scars, hence certain products can provide support for treatment and can be highly beneficial by reducing stress-related skin-picking.^29^

Patients should be encouraged to use dermatologically tested, oil-free and non-comedogenic products that are compatible with skin types, instead of many unproven products on the market. The skin should be gently cleaned twice a day with cleansers that are compatible with the skin pH close to 5.5 without soap. Some of the emollients are cosmeceuticals that contain ingredients such as resveratrol which are useful in acne treatment. Combinations such emollients with cleansers can increase patient compliance and treatment success, especially for those receiving topical isotretinoin and benzoyl peroxide.

It has to be taken into account that the products used for makeup and camouflage should have the same properties. Makeup products should be easy to remove and not close pores. Sun protection is also an important part of treatment since sun exposure can be a triggering factor in some patients. In addition, there is an increase in sun sensitivity, especially in patients who use retinoids (topical and systemic), tetracycline and benzoyl peroxide. The sunscreens to be used for this purpose should be non-comedogenic, oil-free, and easily removable products that do not close pores.

It has been reported a once‐every‐other‐day application of a fixed combination of benzoyl peroxide and isotretinoin accompanying emollients may have the same effect as the daily use combination preparations without the use of emollients. Therefore, it can be suggested that using emollients along with topical treatments can decrease the peeling and irritation of the treatments resulting in increased compliance of patients.^87^

### Maintenance therapy

Adolescent acne responds rapidly to treatment, while adult acne is more resistant and frequent relapses are seen, therefore adult acne is requiring longer maintenance therapy. Topical retinoids and azelaic acid are highly effective products and can be preferred for maintenance therapy, especially in adult female acne.^88^, ^89^

## Conclusion

In conclusion, acne shares different properties with adult and adolescent acne. These differences extend from epidemiology to treatments. Increased awareness of these two subtypes will allow for better management of the disease. Lastly, there are some questions that should be addressed regarding these two subtypes.

1. During adolescence, the female gender is not a risk factor. Why should it be in adult acne?
2. Males are underrepresented in adult acne. What is the protective mechanism?
3. What is the difference in the effect of diet on the two groups?
4. Deep nodules on the chin are a hallmark of adult acne. Is there any histologic study to characterize the lesions?
5. What is responsible for the different facial distribution of acne lesions in adolescent and adult acne?
6. If adult acne is not comedogenic, is it still acne or merely a rosacea-like disease?

These questions seem to shape future studies on acne.

## Financial support

None declared.

## Authors’ contributions

Ömer Kutlu: Approval of the final version of the manuscript; critical literature review; data collection, analysis, and interpretation; effective participation in research orientation; ıntellectual participation in propaedeutic and/or therapeutic management of studied cases; preparation and writing of the manuscript; study conception and planning.

Ayşe Serap Karadağ: Approval of the final version of the manuscript; data collection, analysis, and interpretation; effective participation in research orientation; ıntellectual participation in propaedeutic and/or therapeutic management of studied cases; manuscript critical review; study conception and planning.

Uwe Wollina: Approval of the final version of the manuscript; data collection, analysis, and interpretation; effective participation in research orientation; ıntellectual participation in propaedeutic and/or therapeutic management of studied cases; manuscript critical review.

## Conflict of interest

None declared.
